# A Chameleon and a Sponge: A Qualitative Study on Adaptability in Undergraduate Clinical Medical Students

**DOI:** 10.5334/pme.2209

**Published:** 2026-05-15

**Authors:** Mattias Theys, Daniëlle M. L. Verstegen, Pim W. Teunissen, Emmaline E. Brouwer

**Affiliations:** 1 Department of Educational Development and Research, School of Health Professions Education (SHE), Maastricht University, Maastricht, The Netherlands; 2 School of Health Professions Education (SHE), Maastricht University, Maastricht, The Netherlands; 3 Department of Obstetrics and Gynecology, Maastricht University Medical Center, Maastricht, The Netherlands

## Abstract

**Introduction:**

Adaptability is the capacity to make appropriate responses to changed or changing situations. Whereas clinicians’ adaptability is framed as a vital part of adaptive expertise, clinical medical students may be adaptable even when they are not yet experts. Investigating this early-stage adaptability can reveal how adaptive expertise first begins to take shape. This study examines how undergraduate medical students experience and enact adaptability during their clerkship rotations.

**Methods:**

Through 20 semi-structured interviews with clinical medical students from two universities, in Belgium and The Netherlands, this qualitative study explores how students perceive and demonstrate adaptability.

**Results:**

The study identifies two domains in which students’ adaptability takes shape: their role as future doctors and their role as learners. In these two domains adaptability functions as both a means to navigate clinical complexity and as a process of professional growth. Results highlight a duality: students need adaptability to thrive during rotations while simultaneously engaging in a process of further developing their adaptability.

**Discussion:**

The ongoing interaction between being adaptable and developing adaptability plays a crucial role in clinical education. Previous research shows that adaptive expertise helps explain how learners turn their experiences of adaptability, central to this study, into skills that support their ongoing professional growth. By making visible how students act and develop adaptability in everyday practice, our findings guide reflection on the implications for clinical education and the build of the foundation of later adaptive expertise.

## Introduction

As future professionals, clinical medical students are continuously confronted with new situations: novel clinical encounters, fast-changing rotations or evolving roles during their training. To be adaptable in such contexts is crucial, not only for the students’ development as learners but also for their preparedness as future doctors [1,2].

Adaptability is defined in different ways in the literature, leading to overlap in some definitions with terms like adaptivity and forms of flexibility. We follow Bartone, Krueger [3, p. 537] who define adaptability as: “the capacity to make appropriate responses to changed or changing situations; the ability to modify or adjust one’s behavior in meeting different circumstances or different people.” In medical education, research on adaptability has often been implicit, focusing instead on related constructs such as preparation for future learning or professional identity formation [1,4,5,6], highlighting the importance of learning to use prior experiences to flexibly navigate new challenges and the importance of moments of adaptation in developing an identity as a clinician [7,8,9]. However, what adaptability entails in the context of being a medical student during clinical placements has been underexplored [10] and this fact represents an important gap, since students in the clinical phase often encounter unpredictable environments that demand ongoing adjustments in learning, interaction, and professional identity [11]. By focusing on what adaptability entails to medical students in the clinical phase, we can begin to understand how they are adjusting to these dynamic circumstances.

Insights into adaptability could provide a crucial foundation for understanding learners’ development towards adaptive expertise. Hatano and Inagaki [12] introduce two paths of learning towards two types of expertise: routine expertise and adaptive expertise. Routine expertise is the ability to efficiently perform recurring tasks with limited variability. In their definition, adaptive expertise is the ability to adapt to changing conditions based on conceptual knowledge that can be applied to new and uncertain situations and balancing this application with efficient execution of routine tasks. Adaptive expertise requires the combination of being able to work efficiently and simultaneously being perceptive to changes and possible innovations. The balance between efficient execution and the ability to adapt has been conceptualized by Schwartz, Bransford [13] as the productive tension between efficiency, which supports routine performance, and innovation, which enables flexible responses to novelty. This framing underscores why adaptive expertise is especially relevant in health professions education, where future physicians must master established practices while remaining responsive to new clinical challenges. We follow this line of reasoning and use the term adaptive expertise for the capacity to balance routine performance and innovation and see adaptability as the ability to adapt to changed or changing situations.

Researchers of adaptive expertise have proposed that educational support for the development of adaptive expertise should start during initial training of medical professionals [1,7,14,15,16,17]. Because adaptability is considered a central element of adaptive expertise, this concept offers a lens that connects students’ experiences of adaptability with the long-term capacities of balancing efficiency and innovation required in professional practice. Therefore, this study explores students’ perspectives on adaptability in clinical education, guided by the question: How do undergraduate medical students in the clinical phase of their study perceive their own adaptability in their practice?

## Methodology

### Design

This qualitative study is in line with a constructivist paradigm. The approach acknowledges that reality is socially constructed and constantly developing [18,19]. We used semi-structured interviews as the data-collection method.

### Setting

Two different sites were selected for this study, Catholic University of Leuven in Belgium, and Maastricht University in the Netherlands. We recruited at two different universities to broaden the range of educational approaches and students’ experiences, creating a more diverse participant pool and did not aim for comparison between the sites. Both sites offer 6-year undergraduate medical education programs, including a 3-year Bachelor degree and a 3-year Master’s degree. In the Master’s program, students at both universities rotate through clinical placements in blocks of 6–12 weeks. Differences between the two sites are found primarily in the Bachelor program. In Maastricht, a Problem-Based Learning (PBL) curriculum is in place, meaning students work in small tutorial groups around real-life challenges, guided by tutors [20]. In Leuven, the Bachelor program consists of traditional lectures accompanied by seminars and workshops. Neither program includes adaptability or adaptive expertise as an explicit learning outcome.

### Participants, sampling & recruitment

We recruited participants from the second year of the Master’s program to ensure participants have clinical experience on which to reflect. Participants were convenience sampled. MT recruited participants by introducing the research study in tutorial groups in Maastricht as well as during lectures Leuven. Interested students provided their email address and were contacted by MT with additional information and, if willing to participate, to sign the informed consent, complete a demographic questionnaire and schedule an interview. After the interview, the participants received compensation in the form of a 10-euro gift card. Data on the participant demographics is provided in Table 1.

**Table 1:** Participants characteristics.


PARTICIPANT CHARACTERISTICS	

**Gender (N)**	

*Men*	3

*Women*	17

*Prefer not to disclose*	0

**Age (years)**	

*Median (range)*	23 (23–25)

**Clinical rotation experience (months)**	

*Median (range)*	13 (8–30)

**Site distribution (N)**	

*Maastricht*	8

*Leuven*	12

**Total**	20


### Data Collection

Our interview guide was informed by research on adaptability and adaptive expertise [2,3,12,13,21,22,23,24,25] Experiences involving routines and adaptability were explored, prompting participants to reflect on their own experiences and behaviour. The interview guide can be found in Appendix 1. Throughout the process of data collection, analysis of initial interviews informed prompts in later interviews. 20 Interviews were conducted in Dutch and lasted between 40 and 80 minutes. MT conducted all interviews on campus or online. Interviews were audiotaped and transcribed verbatim.

### Data Analysis

We used thematic analysis in this study [26,27]. We analyzed in an inductive, iterative manner going back and forth through the data. By refining and redefining the themes throughout the process we are claiming interpretive authority [26,28]. Analysis started after the first interviews with memos and familiarization with the data. The concept of information power guided us in evaluating our data as sufficient. The early onset of the analytical process allowed the author team to judge data sufficiency after 16 interviews, based on the quality and specificity of the dialogues resulting in rich interview data [29,30]. We still conducted four remaining, planned interviews as this further enriched our data. During analysis, memo writing assisted in further developing the themes [31]. MT and EB both coded the first 3 interviews and met to discuss their findings and code descriptions. The codebook resulting from these meetings served to code the next interviews, with MT and EB having weekly meetings to discuss further development of the code book and the outline of the different themes throughout the data. These initial themes were discussed with the other authors, DV and PT. Themes were verified by MT and EB, reviewing the data again. Atlas.ti Version 21.1.1.30813 was used to support analysis [32]. MT translated quotes after finishing the analysis, while writing the manuscript.

### Research team and reflexivity

Ethical approval was obtained from Faculty of Health, Medicine and Life Sciences, Maastricht University, Niet-WMO Verplicht Research Ethics Committee (**FHML-REC/2023/112**). The Educational Committee at Catholic University Leuven confirmed no additional approval was necessary. No team member has any personal or professional relationship to the participants. Monthly meetings with all authors assured that the process was closely followed by each team member and interpretations could be challenged or backed up. The research team is made up of four researchers in medical education, each with different areas of expertise. MT is a PhD candidate with a background in educational sciences. DV is a senior researcher with a background in learning psychology and instructional design. EB is a senior researcher with a background in medicine and international medical curricula. PT is a professor of work-based learning in healthcare and a physician working in obstetrics & gynecology. We acknowledge that our positionalities shaped how we interpreted the data, hence why monthly meetings with all researchers present were important to explicitly reflect on the interpretations [33]. Reflections on data were often colored by the different backgrounds within the research team, e.g. instructional design or a profound focus on theory. Rigour was supported through strategies consistent with constructivist qualitative research, including prolonged engagement with the data, starting with data collection and memo writing, and iterative, collaborative interpretation through team meetings to ensure that the findings were credible, trustworthy, and grounded in participants’ meanings [28].

## Results

### Learning to be Adaptable by Constantly Adapting

Our participants indicated that they are developing adaptability by being adaptable. They interpreted adaptability as the general ability to change in response to their contexts. According to the participants, they need the ability to adapt to new situations during their clerkships to function properly. They also feel it is expected by the community of practitioners in clerkships that they both have and further develop this ability. To participants, adaptability encompasses perceiving what a new practice and learning environment requires in order to participate in that environment. A quote by a participant describes this experience: (A3) “You are just constantly a chameleon and a sponge really. A sponge for information you have to take in and a chameleon because you have to constantly adapt to everything and everyone.”

We found that there are two domains in which this duality features in different ways: participants’ role as a future doctor and their role as a learner. Both roles are intertwined and participants act in them simultaneously. Making the distinction allows us to make different experiences visible.

### Becoming a Doctor

The duality of being adaptable and still having to develop the ability to be adaptable is apparent in participants’ roles as future doctors. Their experiences often related to their functioning in the hospital as a doctor-to-be, for instance communicating with patients, taking a history, performing a physical examination or doing administrative work. They perceived adaptability as relevant to their current contribution to healthcare practice as a physician. (B5) “I found the position of being a clerk, I found that to be a privileged position. (…) In your work itself you do have responsibility, and you want to do it [being a doctor] well, but you are always under supervision. Actually, it’s an ideal year to discover everything, to learn a lot.” This quote highlights that students are learning while also adapting to having the responsibilities of a doctor. This adapting to their future doctor role is further divided into two categories, one relating to medical expertise and the other referring to the social and collaborative component of their future role as a doctor.

Participants acknowledged that their knowledge is limited and their perceived absence of knowledge drove adaptability, keeping students on their toes to constantly look up information specific to the cases they encounter in the clinic: (A1) “Of course the medical theory is a very big part and you develop that in two areas. You do that by learning and observing in the workplace and on the other hand by spending time on it in your own time as well, so by studying or if I see something I don’t know, looking it up.” Participants mentioned the importance of developing their medical expertise and that knowing the basics is essential for that development. (B10) “I think that is also the most important thing about the internships: what you know theoretically, putting that into practice and really being able to see the patient yourself, and also noticing that it is not all textbook medicine.” Being adaptable in applying medical content knowledge means, for example, looking up a diagnosis in between shifts to make sure tounderstand the proposed treatment policy or writing down specific details from a case in order to recognize it in the future. It is through the ability to employ current knowledge even in situations where that might be insufficient, and by continuing to develop their medical expertise, that participants develop into their role of doctor-to-be.

Being adaptable in communication and collaboration relates to managing different expectations and working with different colleagues, patients and supervisors, throughout their clerkship period. A participant put it very strongly: (A4) “Communication and collaboration. I think those are also two very important things or core components of a clerkship because for each clerkship you’re somewhere different each time…” Participants mentioned the importance of communication and collaboration for their daily practice in a hospital and they explained they had to adapt to fit in. To be able to participate they had to become part of the workforce in the hospital and not only be there as a student. Participant B3 described this process: (B3) “So it starts – I think – with assessing what [the supervisor] is like. And then I think I usually did that the first week or two to observe a bit: how does he behave towards others? What does he expect from me here? And then afterwards that went by and then it loosened up a bit.” Being adaptable in communication means taking note of contextual requirements, such as meeting supervisors’ expectations, being able to shift your tone when speaking to supervisors or patients, making sure you can articulate a patient’s diagnosis clearly or speaking up if you don’t understand the situation. Being adaptable in collaboration means using the help of the different actors in the ward (e.g. nurses, administrative personnel) to perform as a doctor in the hospital. Another specific example is collaborating under the duress of evaluation, answering to the expectations of supervisors while also being vulnerable enough to ask questions. This requires adapting both attitude and strategy. Some participants indicated the constant adapting of communicative style as a main cause of fatigue at the end of their day. However, participants see adapting communication and collaboration practices as a key aspect of their future self as a doctor: (B1) “So I think mainly what is important is that we become good communicators. Ultimately, that’s the essence of… Or at least one of the most important tasks as a general practitioner.”

### Being a Learner

The duality of being adaptable and still having to develop the ability to be adaptable simultaneously featured in participants’ role of learner during clerkship periods. A participant articulated a first reflection on this topic: (A5) “Okay, I’m starting my clerkship, I’m still a rookie, I have no idea what to do. How am I supposed to learn in this setting?” Adaptability related to finding new ways to (prepare to) learn, to notice and actively seek learning opportunities and to manifest as a proficient learner.

Preparing to learn means anticipating the needs and dynamics ahead of a rotation. A participant described this anticipation: (B3) “I tried to hear from the previous student: how was that rotation? What do they expect from you there? How do they like you to behave?” Participants described trying to find new strategies to get information to prepare for learning in a particular department or even from specific preceptors. Others tried to arrange meaningful learning moments by planning ahead: (A3) “It’s adjusting every day: okay, what do I want to do today? I often try to plan it a week in advance, so that I can email a doctor in time: is it okay if I join you for a few hours that day?” These tactics illustrate adaptability in their role as a learner, being aware of the challenges ahead. Participants also indicate that they learned these strategies along the way, and from peers, showing development in their abilities to be adaptable as learners.

Adapting is also recognizing learning opportunities, trying to make room for themselves as learners and not only being a part of the workforce as this participant stated his thoughts on trying to make room for himself: (A6) “And nowadays I sometimes think: maybe I should push the boundaries a little bit to make the supervisor feel like: I am here for a reason.” Being adaptable in noticing and seeking learning opportunities means daring to speak up when confronted with something they don’t know, asking clarifying questions and being observant. (B12) “You can learn a lot more if you do things yourself, ask to do things. I did a lot of things that I never thought I could have done as a clinical student (…) because that’s where you learn to, yes, kind of stand up for your own, for your learning.” Participants needed to adapt their learning strategies to get the most out of their time during rotations. Participants indicated this adaptation takes time to get right, throughout the clerkship period but also at every new placement, showing both the need to be adaptable to learn, as well as having to keep developing this ability to be adaptable as a learner.

Participants also experienced a need to present themselves as eager learners in a competitive program. Appearing like a proficient learner means making yourself visible in a positive way, e.g., asking questions or trying to anticipate questions from a supervisor. The adaptability is apparent in the constant being on the lookout to present yourself, not as a doubting, insecure individual, but a well prepared, knowledgeable student, eager to learn. Participant A6 mentioned the burden of proof in this case: (A3) “(…) with rotations, you have to prove to a different doctor every day that you have that knowledge, that you are worthy to be there. Because not every doctor always thinks so.” It means adapting the way you present yourself and correctly gauge expectations: (B1) “Problem is that you have to be able to judge very well in what situation that you can and may do that. (…) I found it very difficult to assess what is expected of me. How inquisitive may I be here, then I feel like: I have not been able to prove myself.” Participants felt that to be perceived as eager learners, in every new placement the operationalization of this quality changed depending on the domain, the culture or the supervisor thus requiring adaptation to find the required strategies.

## Discussion

The results of this study warrant reflection on our understanding of student development. In particular, this section explores reflections on the results and theoretical connections between adaptability and professional identity formation and with adaptive expertise theory.

We found that students’ experiences of their adaptability relates to two roles: their role as a future doctor and their role as a learner. Participants invested time and effort in adapting, through which they simultaneously improved their adaptability and developed their own strategies. This duality of participants’ adaptability played out differently in the two roles. As a future doctor, adaptability is about using knowledge and calibrating attitudes in communication and collaboration, which they see as relevant for their future role as a doctor. As a learner, adaptability is about giving yourself the best chance to learn, preparing for it, noticing opportunities and manifesting as a competent learner. Maybe this is a result of hierarchy, wanting to perform before the eyes of someone higher up the ladder, similar to the findings of Vanstone and Grierson [34] who theorized a model of medical students’ strategies of negotiating hierarchy in the workplace. Their model shows that medical students during clerkships adapt and refine strategies and how adaptability is a key part of the students’ socialization process. Additionally, our overarching theme of “learning to be adaptable, by constantly adapting” emphasizes the need for exposure to situations in which students can experience and thus learn this adaptability. The dynamic interplay between acting and developing adaptability underscores the central importance of adaptability in clinical education and points to how it may lay the groundwork for later professional adaptive expertise. Our results suggest that during their clerkships, learners have plenty of exposure to situations that trigger adaptability development, however support for this development is lacking. Support surrounding students’ adaptability could be included in general support mechanisms already in place in workplace learning settings e.g. mentoring or orientation programs, both focusing on affective and cognitive outcomes [35].

The two roles identified in this study, i.e. the “future doctor” and the “learner”, provide meaningful insight into how adaptability is experienced in different situations. Adaptability seems to be part of an ongoing identity development: by adjusting how they engage, how they communicate and how they present themselves, learners are refining their emerging professional identities. The fluidity of students’ roles in adaptability resembles that of professional identity development, students gradually grasping how they are becoming the doctor they want to be while still being in a formal learners’ position [6,36,37]. Kay, Berry [5], O’Doherty, Culhane [38] found that exposure to clinical practice triggers professional identity development. The duality in the overarching theme of this study also highlights the importance of exposure to situations that require adaptability and the opportunities for development this brings with it, both for students’ adaptability and their professional identity. Students’ adaptability therefore reflects not only a response to day-to-day clinical demands but also an ongoing process of trying out, consolidating, and integrating different aspects of who they are becoming as doctors. Furthermore, interactions with the environment are essential for the development of a professional identity; experimentation with new-found roles can help shape this [39,40]. Looking at our results, being adaptable in different ways, according to students’ roles as a learner and as a future doctor, would fit in this development process and be part of the essential foundation of education: transforming the self into new ways of thinking.

In addition to parallels with the professional identity formation literature, our results also warrant reflection relative to recent adaptive expertise research. For example, Kawamura, Harris [41] showed that pediatric residents develop adaptive expertise in communication through “shifts” in understanding patient and family perspectives. Ott, Schwartz [42] found that residents’ hesitation in the operating room should not be seen as incompetence but should be viewed as an opportunity for productive engagement with uncertainty that creates space to learn how to deal with changing conditions. Spafford, Schryer [43] show that uncertainty frequently emerges in novice optometry students’ case presentations, with learners often responding by emphasizing gaps in their knowledge. Across these studies, adaptive expertise is shown to develop through specific moments of adaptability, responding to disruptions in understanding or uncertainty. Our results align with these insights by showing how similar moments of adaptability play a role in students’ contexts and how students actively engage with these moments in everyday practice. Taken together, these studies and our results reinforce the idea that adaptive expertise provides a robust framework for understanding how students transform moments of adaptability, the focus of the present study, into capacities that are essential for professional growth. The descriptions of how adaptability plays out in a students’ reality is crucial to advance research and practice in this area. Echoing the call by Schwartz [44] for education that can foster adaptability, the results of this study can help educators build innovation activities. Understanding how students are adaptable creates opportunities to build towards adaptive expertise as professionals.

Adaptive expertise literature puts forward the importance of the balance between innovation and efficiency [13]. Adaptability is a central aspect of adaptive expertise, being able to adapt to new and innovative ideas. In that light, reflecting on these results on participants’ adaptability, questions the nature of innovation. Clearly, it is not about finding a new method to treat a disease or designing new policies as previous research pointed out that students do not feel this as part of their task [16]. This study shows that for clinical students, innovation means adjusting to something new to them, in relation to learning or the role of being a doctor. Viewed through the lens of adaptive expertise, the adaptability described in our results reflects students’ capacity to function effectively as learners by responding to different learning environments and making the most of learning opportunities. In this way, developing adaptability as a student may contribute to the longer-term development of adaptive expertise in professional settings. By acknowledging adaptability as a learner-focused capacity, students’ experiences with adaptability can become more visible, also to supervisors, as new-to-them is a much broader category of situations.

Lastly, besides theoretical reflections, our results also carry practical implications on the individual and institutional level. The analytical distinctions made in this study can bring forth new ways of thinking about the clerkship period as a period of adaptation, providing input for targeted reflection on the learners’ different roles. This targeted reflection can be structurally incorporated in already existing portfolio structures. Supervisors or mentors can use our results to initiate conversations about students’ adaptability to discuss these experiences. Our results indicate that during this formative phase of medical students’ development, important strategies, tactics, behavioral responses and habits are developed that likely impact the further development of adaptive expertise during later stages of their professional careers. As such, our results can guide dialogue between students and supervisors to start thinking about their own adaptability during their clinical education.

### Future research suggestions

The role of a learner in being adaptable as a student raises questions about the presence of adaptability behaviours in earlier educational phases. These results warrant a look at medical students at the pre-clinical level to further complete the picture of students’ experiences with adaptability. Alternatively, establishing the connection between medical students’ experiences of adaptability and adaptive expertise as established in theory for professionals, expands our understanding of what constitutes expertise.

### Limitations

This study was exploratory in nature, focusing on a specific stage of the medical curriculum and using in-depth interviews to gain insight in the experiences of students. A methodological limitation of this study is the use of convenience sampling, which, while practical within the time constraints of clerkship schedules, may have resulted in a participant group that was more motivated, more reflective, or otherwise unrepresentative of the wider undergraduate clinical student population [44]. While the choice for clinical students was a conscious one, their current immersion in the hospital context made it so that examples used in interviews were mostly focused on their hospital work. Previous pre-clinical or other learning environments may be left underrepresented in our results. Secondly, we have gained limited insight into the influences of the two different educational contexts on the described experiences. While comparison was never our goal, future comparative research could help clarify how specific contextual factors play a role in students’ adaptability and expertise development. Lastly, given that we put adaptability at the forefront of our study because of its relevance in the field of health professions education, other elements, like routine skills, of adaptive expertise that might play a role in a clinical students’ context are not represented.

## Conclusion

This study brings insight in the ways students adapt in different ways, as a future doctor and as a learner. The separation of these roles allows us to better understand the different behaviours described by students. They develop adaptability in anticipation of becoming a doctor, developing medical practical knowledge and the nuances of communicating and collaborating in the hospital. As a learner, students are adaptable in trying to navigate the dynamic environment and still learn by making space and bringing themselves into the limelight. Introducing the categories of being and becoming adaptable further unravels the complex trajectory towards expertise that these students go through, framing adaptability not only as a future competence but as an integral part of their educational journey towards becoming a doctor.

## Additional File

The additional file for this article can be found as follows:

10.5334/pme.2209.s1 Appendix 1. Interview guidelines.
