# Supplementary material for: A Chameleon and a Sponge: A Qualitative Study on Adaptability in Undergraduate Clinical Medical Students
Source: Perspect Med Educ. 2026 May 15;15(1):432–41. doi: 10.5334/pme.2209 (PMC13178604; doi:10.5334/pme.2209)
Supplement: Appendix 1. — Interview guidelines. [file pme-15-1-2209-s1.pdf]

Appendix 1: Interview guidelines

Introductory Questions

1. Why did you choose to study medicine?
2. What does a typical week look like for you?

Questions

1. We are looking for what expertise development means for you as a student. What is essential to you about being an intern?
2. What are the different tasks involved?
3. What do you think it means to be a medical student?
4. What developments in expertise do you think you have already undergone?
5. What does that process look like?
6. In the literature, expertise development is also described as a process with a balance between routine skills on the one hand and skills aimed at adaptation and innovation on the other. How do you see that reflected in your description of the development you are going through?
7. How do you adapt to changing situations?
8. How do you think these skills will be useful to you in the future as a doctor?

Rounding up

1. Were you able to reflect on your own experiences during this interview?
2. Would you like to add something to our conversation?
